# Regulation of Pdx1 by oxidative stress and Nrf2 in pancreatic beta-cells

**DOI:** 10.3389/fendo.2022.1011187

**Published:** 2022-09-15

**Authors:** Sharon Baumel-Alterzon, Donald K. Scott

**Affiliations:** ^1^ Diabetes, Obesity and Metabolism Institute, Icahn School of Medicine at Mount Sinai, New York, NY, United States; ^2^ Mindich Child Health and Development Institute, Icahn School of Medicine at Mount Sinai, New York, NY, United States

**Keywords:** Pdx1, ROS, Nrf2, oxidative-stress, beta-cells, diabetes

## Abstract

The beta-cell identity gene, pancreatic duodenal homeobox 1 (*Pdx1*), plays critical roles in many aspects of the life of beta-cells including differentiation, maturation, function, survival and proliferation. High levels of reactive oxygen species (ROS) are extremely toxic to cells and especially to beta-cells due to their relatively low expression of antioxidant enzymes. One of the major mechanisms for beta-cell dysfunction in type-2 diabetes results from oxidative stress-dependent inhibition of PDX1 levels and function. ROS inhibits Pdx1 by reducing *Pdx1* mRNA and protein levels, inhibiting PDX1 nuclear localization, and suppressing PDX1 coactivator complexes. The nuclear factor erythroid 2-related factor (*Nrf2*) antioxidant pathway controls the redox balance and allows the maintenance of high Pdx1 levels. Therefore, pharmacological activation of the *Nrf2* pathway may alleviate diabetes by preserving Pdx1 levels.

## Introduction

**Figure 1:**
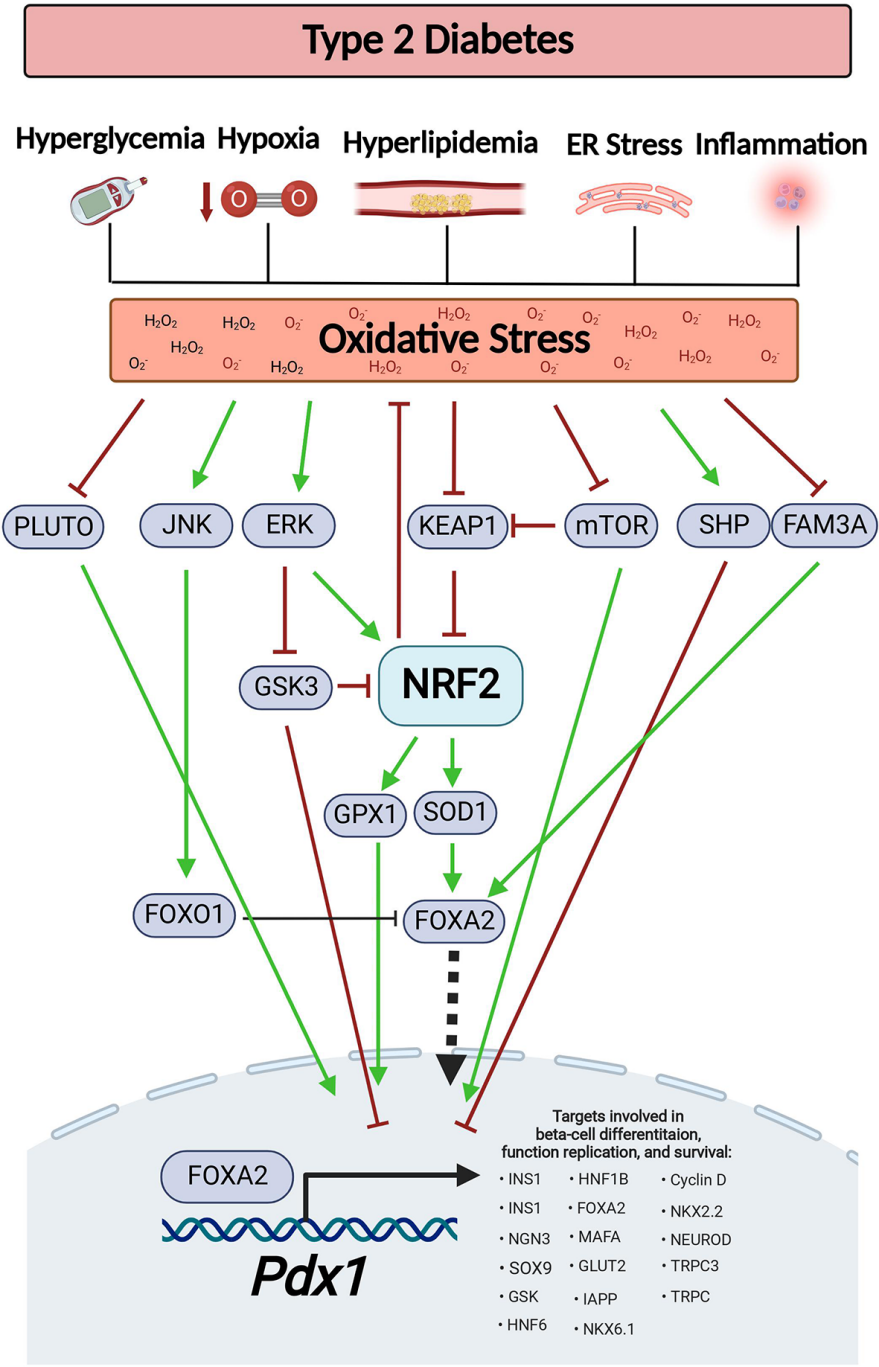
Oxidative stress-dependent pathways affect Pdx1 levels during diabetes. Type-2 diabetes is associated with various pathological conditions that generate ROS, such as hyperglycemia, hyperlipidemia, hypoxia, inflammation, and ER stress. This results in oxidative stress. Oxidative stress reduces the expression level of *PLUTO* lncRNA, stimulates JNK-dependent FOXO1 activation, inhibits mTOCR signaling, increases SHP expression and reduces FAM3A levels, all of which results in decreased PDX1 levels. On the other hand, in order to maintain ROS at appropriate levels, oxidative stress also serves as a signal for Nrf2 activation by inhibition of Keap1 and by activation of the ERK signaling. This results in increased expression of the Nrf2 target genes, *Sod1* and *Gpx, which* restore PDX1 levels. This figure was generated using BioRender.com.

Since its first introduction into the scientific community in 1945, reactive oxygen species (ROS) have been the focus of numerous studies (1). ROS are defined as oxygen-derived atoms or molecules that possess one or more unpaired electrons, making them highly reactive (2). While the cytoplasm, cell membrane, endoplasmic reticulum (ER) and peroxisome are capable of producing ROS, up to 90% of cellular ROS (composed mostly of *H*_2_ *O*_2_ and $O_{2}^{-}$ ) are generated in the mitochondria due to incomplete reduction of oxygen to water in the electron transport chain (ETC) (3). High levels of ROS are extremely toxic to the cell, leading to DNA breaks, lipid peroxidation, protein aggregation, protein denaturation and protein fragmentation (4). Therefore, most cells are equipped with a battery of antioxidant genes that have the power to neutralize this threat, maintaining a fine equilibrium between ROS production and an antioxidant defense. In cases where ROS levels exceed the cell’s ability to detoxify it and the equilibrium is breached, the cell enters into a pathophysiological condition called “oxidative stress” (5). Oxidative stress takes part in the etiology of many diseases, contributing to the initiation and progression of cancer, vascular-related diseases, respiratory diseases, neurodegenerative disorders, digestive diseases, kidney diseases, chronic inflammatory disorders, aging and diabetes (5–7). Diabetes is linked to various cellular stresses that generate ROS, such as hyperglycemia, hyperlipidemia, hypoxia, inflammation, and ER stress (8, 9) (**Figure 1**). Indeed, pancreatic islets of type-2 diabetic patients often present increased levels of oxidative stress markers (such as oxidative DNA damage marker, 8-hydroxy-2′-deoxyguanosine) and this, at least in part, is due to the fact that beta-cells express low levels of several antioxidant genes, making them highly vulnerable to increased ROS levels (7, 10). In line with that, high ROS levels reduce functional beta-cell mass by increasing beta-cell apoptosis, reducing beta-cell proliferation, and damaging beta-cell function (7). Therefore, and perhaps as a compensatory mechanism, beta-cells protect themselves against oxidative stress by activating the nuclear factor erythroid 2-related factor (*Nrf2*) antioxidant signaling pathway. This activation is performed by ROS which oxidize critical cysteine in the NRF2 inhibitor, KEAP1, thus inhibiting NRF2 degradation (7, 11). Remarkably, apart from providing protection against oxidative stress, NRF2 regulates beta-cell mitochondrial biogenesis and activity, provides anti-inflammatory effects, promotes beta-cell function and stimulates beta-cell proliferation (7, 11).

Beta-cells express a unique set of key marker genes that define their identity and function by contributing to the expression and secretion of insulin (12). One of these genes is the duodenal homeobox 1 (*Pdx1*), a protein that plays many distinct roles in the life of a beta-cell, including involvement in beta-cell differentiation, beta-cell function, beta-cell survival and beta-cell proliferation (13–18). To do so, PDX1 controls the expression of other genes that play important roles in the beta-cell fate. These genes include insulin (*Ins1*, *Ins2*), neurogenin 3 (*Ngn3*), SRY-box transcription factor 9 (*Sox9*), hepatocyte nuclear factor 6 and 1b (*Hnf6, Hnf1b*), forkhead box protein a2 (*Foxa2*), V-Maf musculoaponeurotic fibrosarcoma oncogene homolog A (*MafA*), NK2 homeobox 2 (*Nkx2.2*), neurogenic differentiation (*NeuroD*), solute carrier family 2 member 2 (Glut2, *Slc2a2*), glucokinase (*Gck*), islet amyloid polypeptide (*Iapp*), NK6 homeobox 1 (*Nkx6.1*), cyclin D1 (*Ccnd1*), cyclin D2 (*Ccnd2*), and the transient receptor potential cation channel family 3 and 6 (*Trpc3,6*) (13, 16, 17, 19). In agreement with these findings, homozygous *Pdx1* knockout (Pdx1^-/-^) mice exhibit pancreatic agenesis while heterozygous *Pdx1* knockout (Pdx1^+/-^) mice present with beta-cell ER stress, beta-cell apoptosis, impaired insulin secretion, decreased beta-cell proliferation and reduced beta-cell mass (20–25). Additionally, tamoxifen-inducible beta-cell specific *Pdx1* deletion in adult mice results in hyperglycemia and transdifferentiation of beta-cells into alpha-like cells (26). In humans, 5% of diagnosed type-2 diabetes patients present with genetic defects in *PDX1* and mutations in *PDX1* define a subset of maturity-onset diabetes of the young 4 (MODY 4) (27, 28).

Defective expression of *Pdx1* has been documented in diabetes and this is attributed to oxidative stress (9, 29–31). Beta-cell specific deletion of *Nrf2* in mice under diabetogenic situation results in increased oxidative stress and reduced Pdx1 expression. Conversely, activation of NRF2 using genetic or pharmacological approaches increases *Pdx1* expression (11). These findings suggest that the NRF2 pathway is essential for preserving PDX1 levels, thus contributing to the maintenance of functional beta-cell mass. In this review, we aim to bring together all the current knowledge about the effect of physiological changes in redox balance on beta-cell fate *via* regulation on PDX1 levels.

## Regulation of PDX1 levels by ROS during embryonic beta-cell development

During embryogenesis, *Pdx1* is expressed in two waves. The first wave begins at early stages of pancreatic development (E8.5 in mouse, gestational week 4 in human), with the appearance of dorsal and ventral foregut endoderm buds that later transform into a fully developed pancreas. At that stage, PDX1 is detected in the pancreas epithelium (13, 32–34). Mutations and deletions of *Pdx1* during the first wave results in pancreas agenesis in both mice and humans. Accordingly, *Pdx1* deletion does not affect the development of the endoderm buds. Rather, deletion of *Pdx1* inhibits the morphogenesis of the buds creating defects in the development of pancreatic epithelium (13, 20, 35, 36). Interestingly, the appearance of early premature insulin and glucagon positive cells in cells that lack PDX1 suggests that unlike in the second wave of *Pdx1* expression, the first wave of *Pdx1* expression is not involved in the development of endocrine cells (34, 36).

At the second wave of *Pdx1* expression, which occurs at late gestation (beginning at E13.5 in mouse, gestational week 12 in human), PDX1 stimulates the expression of another developmental factor, *Ngn3*. NGN3 levels, which significantly increase in mouse at E13.5 and reach maximal levels at E15.5, are responsible for the specification of endocrine progenitors into endocrine cells that will later form the pancreatic islets (13, 37, 38). Inducible *Pdx1* deletion at these ages blocks the formation of acinar cells and islets in mouse embryos (35). Additionally, beta-cell specific *Pdx1* deletion increases glucagon and somatostatin positive cells at the expense of insulin positive cells resulting in diabetes (34). Following the second wave of *Pdx1* expression, PDX1 levels remain constant towards and during adulthood (in healthy non-diabetic settings). In adults, PDX1 is restricted to beta-cells where it works to maintain beta-cell maturity and function (13, 33).

Some levels of ROS are necessary for normal cellular function. For example, moderate levels of ROS (up to ~100 nM H_2_O_2_) are needed for stem cells to maintain their ability to go through cell differentiation and to keep their “stemness”. The underlying mechanism involves the oxidation of tyrosine and cysteine residues which activates protein kinases, protein phosphatases, and signaling factors that take part in cell differentiation (39). This might explain the formation of moderated ROS levels (starting at E12.5 and peaking at E15.5) by NADPH oxidase 4 (*Nox4*) activity during the development of embryonic mouse pancreas (40). Nevertheless, accumulation of higher ROS levels (above ~100 μM H_2_O_2_) may lead to cell senescence or death (39). Furthermore, prompt increase of oxidative stress may pose a risk on normal pancreatic development since PDX1 levels are negatively regulated by ROS (for further details on the mechanisms see **Figure 1** and **Table 1** below) (41–45). This highlights the need to maintain ROS at appropriate levels to support pancreatic development and differentiation without damaging the cells. Surprisingly two NRF2 antioxidant target genes, catalase (*Cat*) and superoxide dismutase 1 (*Sod1*), are upregulated during pancreatic development (40). This suggests that NRF2 helps maintaining the required redox balance in the embryonic pancreas, a theory that has not been tested yet. In support of that, SOD1 stimulates *Pdx1* expression by increasing the expression of Forkhead Box Protein A2 (*Foxa2*), one of the main *Pdx1* transcriptional regulators (46). SOD1 can also support *Pdx1* transcription by increasing H3 acetylation and H3K4 methylation on the *Pdx1* promoter, thus providing an open active chromatin (46). Intriguingly, during pancreatic development, ROS activates the ERK signaling pathway which can further activate the NRF2 pathway and increase *Pdx1* expression, resulting in differentiation of endocrine progenitors into beta-cells (47–49). Overall, these findings suggest that by maintaining normal redox balance, the NRF2 pathway supports appropriate PDX1 levels that promote normal pancreatic development.

## Regulation of PDX1 levels by ROS during postnatal ages and adulthood

After birth, both mouse and human neonates exhibit a sharp burst of beta-cell proliferation. This peak of proliferation, which is temporary, substantially declines with age (50). Weaning is believed to be the main trigger for the loss of beta-cell proliferative capabilities, and it coincides with the gradual maturation of beta-cells (51, 52). Mechanistically, the transition from fat-rich maternal milk to carbohydrate-rich diet inhibits the proliferative factor, mammalian target of rapamycin complex1 (mTORC1) and activates 5’-adenosine monophosphate–activated protein kinase (AMPK), which promotes beta-cell maturity. On the other hand, continuous supplementation of milk-fat rich diet to mice during weaning into adulthood maintains high mTORC1 levels and beta-cell proliferation (53). Consistent with these findings, beta-cell specific deletion of *mtorc1* in mice leads to impaired beta-cell proliferation, reduced beta-cell survival and decreased beta-cell mass (54), while activation of mTORC1 stimulates mouse beta-cell proliferation by increasing cyclin D2 expression (55). Interestingly, branched chain amino acids can activate the mTORC1-Rab1A axis to maintain PDX1 protein stability and increase its nuclear localization (56, 57). Moreover, beta-cell-specific overexpression of kinase-dead mTOR mutants results in decreased *Pdx1* mRNA and protein levels (58). These findings link mTOR-stimulated beta-cell proliferation during postnatal ages with PDX1 activity. Oxidative stress and conditions that generate ROS such as ER stress and hypoxia, can inhibit mTOR signaling (59). In postnatal Akita mice, ER stress inhibits mTORC1 leading to reduced beta-cell proliferation, as well to decreased PDX1 protein levels and downregulation of PDX1 target genes. However, restoration of mTORC1 activity in these mice did not affect PDX1 protein levels, suggesting that ER stress affects PDX1 levels by mTORC1-independent mechanisms (60). Although the exact mechanism by which mTORC1 regulates *Pdx1* is unknown, mTORC1 can control NRF2 levels by p62-dependent degradation of its inhibitor, KEAP1 (7, 61), suggesting that NRF2 might be involved.

PDX1 binding sites are found in several genes that are associated with cell replication, such as *Nasp, Bard1, Mnx1*, and *Mcm7* (17). This suggest that PDX1 on its own can stimulate beta-cell proliferation. Accordingly, overexpression of *Pdx1* in primary rat islets increases beta-cell proliferation by upregulation of cyclin *D1* and *D2* expression (16), which are essential for beta-cell proliferation during postnatal ages (62, 63), over-nutrition (64, 65), and pregnancy (66). Interestingly, oxidative stress reduces cyclins *D1* and *D2* expression while activation of the NRF2 antioxidant pathway increases cyclin D1 (9, 11). On the other hand, mice expressing mutated PDX1 develop diabetes at weaning concomitantly with reduced beta-cell proliferation and beta-cell area (17). Thus, regulation of redox-balance is important for PDX1-stimulated beta-cell proliferation.

During adulthood, PDX1 switches roles and takes part in beta-cell identity and maturation. For example, PDX1 controls insulin gene transcription by forming a transcriptional activation complex with neuronal differentiation 1 (*Neurod1*) and by upregulation of *MafA* and *Ngn3* expression, which are needed for insulin transcription (13). MAFA can further enhance glucose-stimulated insulin secretion (GSIS) to maintain glucose homeostasis (67). PDX1 can also upregulate the expression of other factors that are necessary for GSIS, such as *Glut2* and glucokinase (28). Indeed, two missense mutations in the *Pdx1* transactivation domain inhibit GSIS (68). Lastly, as previously mentioned, PDX1 positively regulates the expression of various beta-cell identity genes (13, 16, 17, 19). This might explain why beta-cell specific *Pdx1* deletion results in an altered transcriptional profile that resembles alpha-like cells. This includes downregulation of *Ins1* and *Glut2* genes while upregulation of *Gcg* (glucagon) and *MafB* (26). These findings suggest that physiological situations that increase oxidative stress may inhibit PDX1 from maintaining beta-cell identity and function, and eventually lead to diabetes (as described in part 4).

## Regulation of PDX1 levels by ROS in beta-cells during diabetes

One of the major mechanisms for beta-cell dysfunction in type-2 diabetes involves the inhibition of *Pdx1* by oxidative stress (41–44). For example, chronic exposure of syrian hamster islet cell line HIT-T15 to high glucose concentrations reduces *Pdx1* mRNA and protein levels (44, 69). Additionaly, type-2 diabetic rodent models, such as db/db mice as well as mice and rats fed on high fat diet (HFD), display reduced *Pdx1* mRNA and/or protein expression as well as decreased PDX1 nuclear localization (53, 70–72). Similarly, beta-cell specific deletion of Nrf2 in mice fed on HFD results in increased oxidative stress, reduced *Pdx1* mRNA levels and inhibition of PDX1 translocation into the nucleus (11). Conversely, treatment of obese diabetic C57BL/KsJ-db/db mice or Zucker diabetic rats with antioxidant agents restores PDX1 nuclear localization and PDX1 transcriptional activity (73, 74). Likewise, overexpression of glutathione peroxidase 1 (*Gpx1*), a Nrf2 antioxidant target gene, increases *Pdx1* mRNA and protein levels in mouse beta-cells (75).

The mechanisms behind ROS-dependent reduction of PDX1 levels vary (**Figure 1** and **Table 1**). For example, oxidative stress can lead to activation of forkhead box protein O1 (FOXO1) by c-Jun N-terminal kinase (JNK) or by acetylation of FOXO1. As a result, FOXO1 goes through phosphorylation, nuclear translocation and subsequent activation (76, 88). FOXO1 then mediates inhibition of FOXA2-transcriptional activation of *Pdx1* and inhibits PDX1 nuclear translocation (76, 89–91). Moreover, islets from mice fed on HFD exhibit nuclear exclusion and reduced activity of FOXA2 (92) and db/db mouse islets show reduced levels of family with sequence similarity 3 member A (FAM3A), a factor that upregulates PDX1 levels *via* activation of CaM-FOXA2 pathway (85). Oxidative stress can reduce PDX1 levels by additional mechanisms. For example, during conditions that generate ROS, such as high glucose concentrations, ER stress and treatment with streptozotocin (STZ) (7), orphan nuclear receptor small heterodimer partner (SHP) downregulates Pdx1 at the mRNA levels (78, 82, 84). In addition, mutations in *Klf11* transcription factor which are associated with maturity-onset diabetes of the young 7 (MODY7), lead to promoter repression of the antioxidant gene catalase 1 (*Cat1*), and to a reduction in *Pdx1* transcription in beta-cells (93, 94). Saturated fatty-acids, such as palmitic-acid, can stimulate the production of H_2_O_2_ in beta-cells, leading to beta-cell death (95). Interestingly, recent findings show that incubation of beta-cells with palmitic acid leads to sequestration of PDX1 into stress-granules. This sequestration prevents PDX1 from translocating to the nucleus and transcribing its target genes. Moreover, inhibition of the stress-granules formation in HFD fed mice results in increased PDX1 nuclear localization and improved glucose tolerance as well as GSIS (81).

**Table 1:** Ros-dependent mechanisms that reduce Pdx1 levels and activity during diabetes.

Diabetic situations associated with oxidative stress	Mechanisms	Reduced Pdx1 RNA levels	Reduced PDX1 protein levels	Reduced PDX1 nuclear localization	Reduced PDX1 transcriptional activity	Citations
**Hydrogen peroxide (H_2_O_2_)**	• Phosphorylation of PDX1 Serine 61/66 by GSK3. • Reduced H3 and H4 histone acetylation in *Pdx1* promoter. • Activation of FOXO1 inhibits FOXA2.	X	X	X		(75–77)
**Chronic exposure to high glucose concentration**	• Hypermethylation at CpG sites on *Pdx1* promoter. • Phosphorylation of PDX1 Serine 268 by Gsk3. • Reduced PDX1-P300 interaction. • Reduced *Pdx1* and *p300* expression by SHP.	X	X		X	(44, 53, 69, 78–80)
**Palmitic acid**	• Sequestration of PDX1 into stress-granules in a PI3K/EIF2α dependent manner.			X		(81)
**Streptozotocin (STZ)**	• Reduced *Pdx1* expression by SHP.	X				(82)
**High fat diet (HFD)**	• Reduced PDX1-CHD4 interaction.	X		X	X	(70, 71, 83)
**ER stress**	• Reduced *Pdx1* expression by SHP.		X			(60, 84)
**db/db diabetic mice**	• Phosphorylation of PDX1 Serine 269 by GSK3. • Reduced *Fam3a* expression inactivates CaM-FOXA2 pathway.	X	X	X		(53, 72, 73, 85)
**Type 2 diabetic human islets**	• Hypermethylation at CpG sites on *PDX1* promoter. • Reduced *PLUTO* lncRNA. • Reduced PDX1-CHD4 interaction. • Reduced expression of *p300*. • Reduced PDX1-SWI/SNF interaction.	X			X	(79, 80, 83, 86, 87)

At the post-transcriptional level, oxidative stress increases activity of glycogen synthase kinase 3 (GSK3), a known NRF2 inhibitor, which then phosphorylates PDX1 serine 61 and/or serine 66 resulting in PDX1 protein degradation (7, 77). Additionally, INS1e rat beta-cell-like insulinoma cells and human islets chronically exposed to high glucose display GSK3-mediated phosphorylation of PDX1 serine 268, resulting in PDX1 degradation and reduced expression of PDX1 target genes (53). Oxidative stress also reduces H3 and H4 histone acetylation in the *Pdx1* promoter, leading to transcriptional silencing due to tightly packed chromatin (75). Islets from type-2 diabetic donors show higher methylation status at ten CpG sites on the *PDX1* promoter. This is associated with reduced *PDX1* mRNA levels. The same phenomenon is observed in rat insulinoma beta-like cells (INS 832/13) incubated chronically at high glucose concentrations (19, 79). Additionally, treatment of human stem cells with the DNA methylation inhibitor, 5-aza-2′-deoxycytidine, increases PDX1 nuclear levels (19, 96). Human and mouse pancreatic islets express hundreds of long non-coding RNAs (lncRNAs), some of which are playing important roles in beta-cell differentiation and function. One of the most characterized ones is a beta-cell specific lncRNA called *PLUTO*, which positively regulates *PDX1* transcription. *PLUTO*, exhibits a marked reduction of expression in islets from type-2 diabetic donors, positioning it as another mechanism for reducing PDX1 levels under ROS-associated pathological conditions (86).

Apart from affecting PDX1 abundance, conditions associated with increased ROS can affect PDX1 transcriptional activity by targeting coactivator complexes associated with PDX1 (97). For example, decreased interaction between PDX1 and the chromodomain helicase DNA-binding 4 (CHD4) ATPase subunit of the NuRD complex is observed in both islets of type-2 donors and in mice fed on HFD (83). Similarly, in human type-2 diabetic beta-cells, there is a significant reduction in PDX1 binding to the ATP-dependent SWI/SNF chromatin remodeling complex, a complex that is needed for pancreas development and beta-cell identity (87, 98). PDX1 also interacts with histone acetyltransferases p300 and CBP (p300/CBP) to stimulate expression of PDX1 target genes, including insulin (99). INS-1E cells incubated in high glucose concentrations and islets from type-2 donors display reduced levels of p300 due to protein degradation (80), a situation that may hamper PDX1 transcriptional activity.

Based on their ability to reduce hyperglycemia and body weight, the U.S. food and drug administration (FDA) approved the use of Glucagon-like peptide-1 receptor (GLP-1R) agonists as a treatment for type-2 patients (100). Interestingly, treatments of islets from a rat model of intrauterine growth retardation (IUGR) or from a “catch up growth” Wistar rat model with the GLP-1R agonists, Exendin 4 and Liraglutide, increase Pdx1 transcription by increasing H3 histone acetylation, increasing H3K4me3 levels and by reducing H3K9me2 levels in Pdx1 promoter (101, 102). Additionally, GLP-1 itself stimulates PDX1 nuclear translocation *via* cAMP-dependent PKA pathway and activates NRF2 through the PKA-dependent ERK activation pathway, suggesting that NRF2 might be involved in this regulation (7, 103).

## Concluding remarks

To conclude, published data indicate that alteration in redox-balance leads to dysregulated PDX1 levels and activity, which can result in diabetes. Expression of several NRF2 target genes, as well as treatment with CDDO-Me, an NRF2 pharmacological activator, maintain PDX1 abundance by reducing oxidative stress (11, 40, 75, 104). These findings suggest that activation of the NRF2 pathway may alleviate diabetes by preserving PDX1 levels. Additional studies are needed to further explore the role of NRF2 in maintaining PDX1 levels during embryonic, postnatal and adult life. Interestingly, CDDO derivatives were also shown to contribute to the maintenance of functional beta-cell mass by promoting beta-cell proliferation, reducing beta-cell oxidative damages, increasing islet cell viability, improving insulin content, stimulating insulin secretion and reducing secretion of proinflammatory cytokines (7, 11). Furthermore, treatment of db/db and streptozotocin-induced diabetic mouse models with CDDO derivatives improves diabetes outcome (105–107) and these compounds have been tested under several clinical trials to improve chronic kidney disease in diabetic patients (7).This places NRF2 as a potential therapeutic target for type-2 diabetes.

## Author contributions

SB-A designed content, wrote text, and produced the figure. DKS reviewed and edited the manuscript. All authors contributed to the article and approved the submitted version.

## Funding

This work was supported by the National Institutes ofHeath/The National INstitute of Diabetes Digestive and Kidney Diseases R01DK114338 andR01DK130300 (to DKS) and K01128387 (to SB-A).

## Conflict of interest

The authors declare that the research was conducted in the absence of any commercial or financial relationships that could be construed as a potential conflict of interest.

## Publisher’s note

All claims expressed in this article are solely those of the authors and do not necessarily represent those of their affiliated organizations, or those of the publisher, the editors and the reviewers. Any product that may be evaluated in this article, or claim that may be made by its manufacturer, is not guaranteed or endorsed by the publisher.
